# Remarkable thermal conductivity enhancement in Ag—decorated graphene nanocomposites based nanofluid by laser liquid solid interaction in ethylene glycol

**DOI:** 10.1038/s41598-020-67418-3

**Published:** 2020-07-03

**Authors:** M. C. Mbambo, S. Khamlich, T. Khamliche, M. K. Moodley, K. Kaviyarasu, I. G. Madiba, M. J. Madito, M. Khenfouch, J. Kennedy, M. Henini, E. Manikandan, M. Maaza

**Affiliations:** 1 UNESCO-UNISA Africa Chair in Nanosciences-Nanotechnology, College of Graduate Studies, University of South Africa, Muckleneuk Ridge, PO Box 392, Pretoria, South Africa; 2 Nanosciences African Network (NANOAFNET), iThemba LABS-National Research Foundation, 1 Old Faure Road, Somerset West 7129, PO Box 722, Somerset West, Western Cape Province South Africa; 3 Discipline of Physics, School of Chemistry and Physics, University of KwaZulu-Natal, Durban, South Africa; 4 National Isotope Centre, GNS Science, PO Box 31312, Lower Hutt, 5010 New Zealand; 5 School of Physics and Astronomy, The University of Nottingham, University Park, Nottingham, Nottingham, NG7 2RD UK; 6 Department of Physics, Thiruvalluvar University College of Arts and Science, Thennangur Village, Vandavasi Taluk, Tiruvannamalai, Tamil Nadu 604408 India

**Keywords:** Energy science and technology, Materials science, Nanoscience and technology

## Abstract

We report on the synthesis and enhanced thermal conductivity of stable Ag-decorated 2-D graphene nanocomposite in ethylene glycol based nanofluid by laser liquid solid interaction. A surfactant free nanofluid of Ag nanoparticles anchored onto the 2-D graphene sheets were synthesized using a two-step laser liquid solid interaction approach. In order to understand a pulsed Nd:YAG laser at the fundamental frequency (λ = 1,064 nm) to ablate Ag and graphite composite target submerged in ethylene glycol (EG) to form AgNPs decorated 2-D GNs-EG based nanofluid. From a heat transfer point of view, it was observed that the thermal conductivity of this stable Ag-graphene/EG is significantly enhanced by a factor of about 32.3%; this is highest reported value for a graphene based nanofluid.

## Introduction

Following the discovery of graphene by Geim and Novoselov^1^, the field of graphene related science ushered new areas of science and potential technologies and several studies were carried out on different aspects of these peculiar 2-D carbon nanostructures. Graphene is a flat monolayer of carbon atoms or a thin layer of pure carbon tightly packed into two-dimensional (2D) honeycomb lattice. Early investigations of graphene demonstrated that this material exhibits extraordinary physical and chemical properties such as significantly higher values of thermal conductivity, electrical conductivity, high room temperature carrier mobility and lateral quantum confinement amongst others. These properties confer graphene and graphene based nanocomposites numerous potential technological applications such as super capacitors, organic solar cells, photovoltaics, fuel cells, and inter alia^2,3,4,5,6,7^. In terms of heat transfer, the thermal conductivity of graphene is demonstrated to be within the range of 3,000–5,000 Wm^−1^ K^−1^ at room temperature which is an exceptional figure when compared to the thermal conductivity of pyrolytic graphite (2000 W m^−1^ K^−1^ at room temperature), and surpasses even the highest value of natural diamond (895–1,350 Wm^−1^ K^−1^) as well as that of standard Cu (384.1 Wm^−1^ K^−1^). Hence, graphene would be an ideal candidate for use in nanofluids applications^8,9,10,11,12^, especially when decorated with additional metallic nanoparticles as will be demonstrated in this contribution.

**Figure 1:**
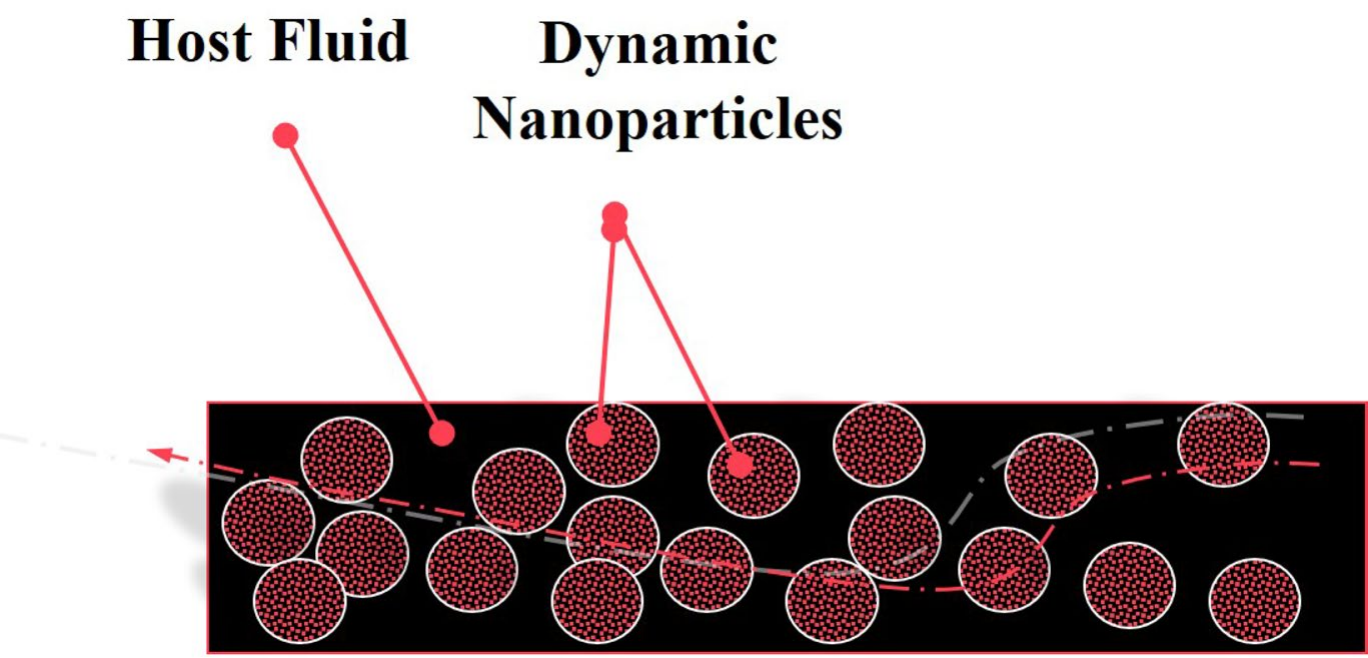
Universal configuration of a nanofluid consisting of nanoscaled particles in suspension in a host standard fluid.

**Figure 2:**
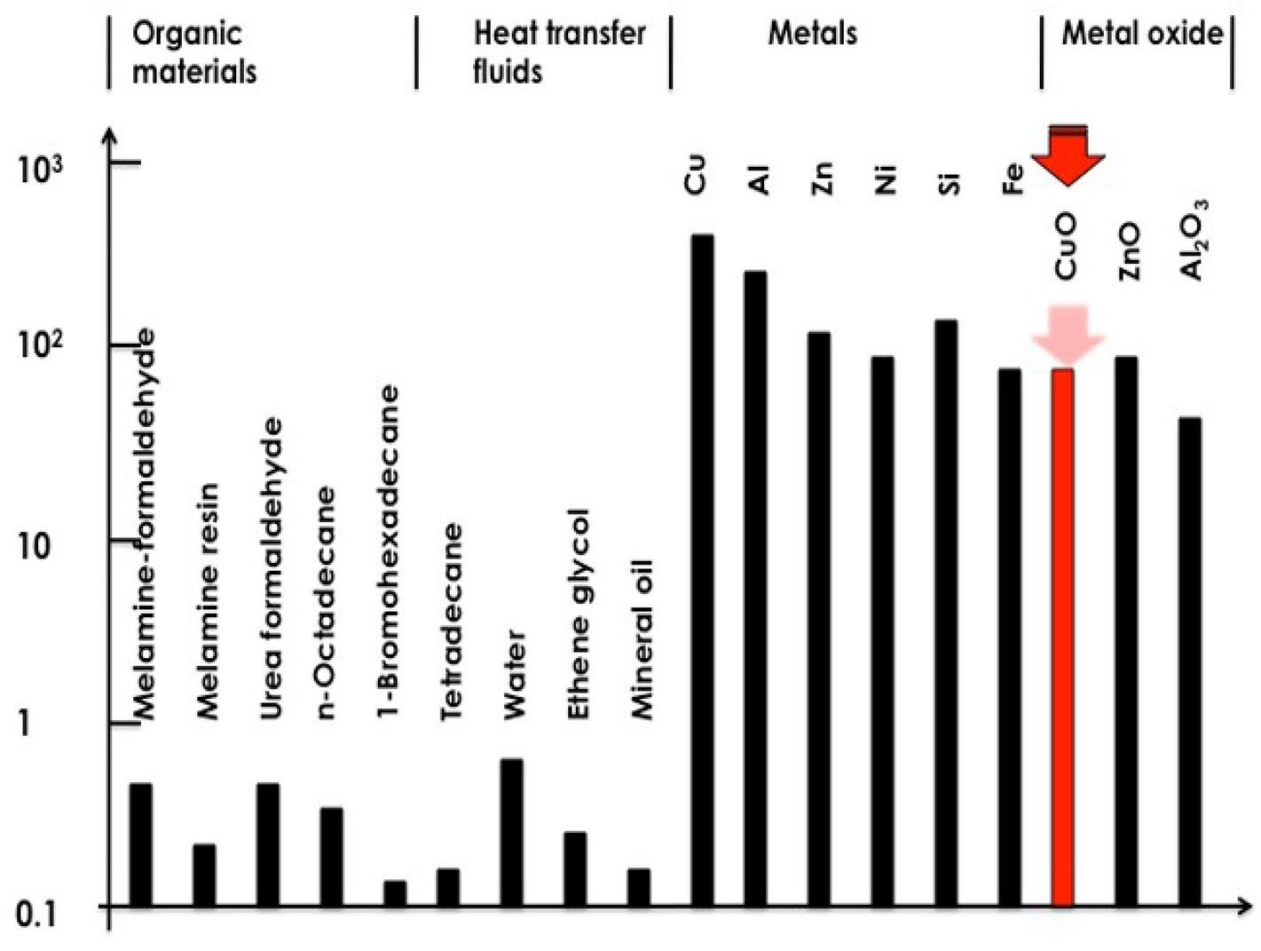
Comparative scale of thermal conductivity of various materials: Organic materials, standard heat transfer fluids, metals and their oxides.

As shown in Fig. 1, nanofluids are a form of molecular fluids consisting of a uniform dispersion of nanoparticles in a traditional coolant host fluid such as H_2_O, oil or ethylene glycol (EG) amongst others. Figure 2 reports a comparison between the thermal conductivity of several organic materials, standard heat transfer fluids (water, ethylene glycol, mineral oil) metals and metal oxides. As one can observe, the thermal conductivity of a standard heat transfer fluid is, generally, lower than < 1 Wm^−1^ K^−1^ at room temperature whilst that of metals and their corresponding oxides are two to three orders of magnitudes higher. Hence, the mixture of such metallic or their oxides nanoparticles in standard coolant host fluid in a form of a nano-suspension would induce a significant enhancement in the thermal conductivity of the nanofluid. Such an enhancement has been predicted theoretically by Batchelor and O’Brien in 1977 and Hamilton, Grosser et al*.,* as early as 1962^8,9,10^. The thermal conductivity of a nanofluid was predicted to be^8,9,10^.

1 $$ {\text{K}}_{{{\text{eff}}}} /{\text{k}}_{{\text{f}}} = [{\text{k}}_{{\text{p}}} + ({\text{j}} - {\mathbf{1}}){\text{k}}_{{\text{f}}} - \, ({\text{j}} - {\mathbf{1}}){\text{h}}({\text{k}}_{{\text{f}}} - {\text{k}}_{{\text{p}}} )\left] / \right[{\text{k}}_{{\text{p}}} + \, ({\text{j}} - {\mathbf{1}}){\text{k}}_{{\text{f}}} + {\text{h}}({\text{k}}_{{\text{f}}} - {\text{k}}_{{\text{p}}} )] $$

2 $$ {\text{K}}_{{{\text{eff}}}} /{\text{k}}_{{\text{f}}} = [ \, {\mathbf{1}} - {\text{h}} + {\text{h}}({\text{k}}_{{\text{f}}} /{\text{k}}_{{\text{p}}} )] {\text{for sphericityj}} = {\mathbf{1}} $$

where K_eff_/k_f_ is the enhancement of the thermal conductivity of the nanofluid, k_f_, k_p_ are the thermal conductivity of the host fluid and the nanoparticle**s** respectively, η being the volume concentration and φ is the sphericity reflecting the shape anisotropy of the nanoparticles. Following the pioneering experimental work of Choi et al*.,*^11^, such an enhancement in thermal conductivity in various nanofluids was confirmed although prepared by different methodologies^12,13,14,15,16,17,18,19,20,21,22,23,24,25,26,27,28^ including direct evaporation, submerged arc nanoparticle synthesis, laser ablation, microwave irradiation, polyol process and phase-transfer. Various nanofluids consisting of nano-scaled Ag, CuO, Al_2_O_3_, TiO_2_ and SiO_2_ in various host fluids^11,12,13,14,15,16,17,18,19,20,21,22,23,24,25,26,27,28^ have further validated this enhanced thermal conductivity reaching values varying from 10 to 70%. However, the highest enhancements, generally, were observed in shape anisotropy nanosystems such as Ag nanorods^27,28^.

The originality of this contribution lies in investigating for the first time the thermal transfer enhancement of a nanocomposite of silver nanoparticles (Ag NPs) decorated 2-D graphene nanosheets (GNs) dispersed in ethylene glycol (EG). In the rest of the manuscript, a ternary-composite nanofluid is labeled as AgNPs-GNs-EG. The rationale behind such an approach is that the 2-D nature of graphene sheets would assist in ensuring the percolation threshold at lower volume concentrations in addition to their elevated intrinsic thermal transfer property combined with their homogeneous dispersion in polar host liquids^29,30,31^. The Ag nanoparticles (Ag NPs) decorating the 2D GNs carpets would act as a facilitating heat medium transfer between the host liquid (EG) and the graphene nanosheets (GNs). In addition, the agglomeration of the (Ag NPs) is likely to be minimized if they are anchored onto the GNs. Hence, in view of validating such a concept, a ternary nanofluid was engineered by pulsed laser ablation in two steps phases: (i) producing graphene sheets first, and then (ii) producing silver nanoparticles. Both are fabricated in the same ethylene glycol host fluid. The measured thermal conductivity of AgNPs-GNs-EG has been found to be to be superior than that exhibited by pure Ag NPs-EG or pure GNs-EG nanofluids^32,33,34,35,36,37^.

## Experiments, results and discussion

### Materials and nanofluid preparation

**Figure 3:**
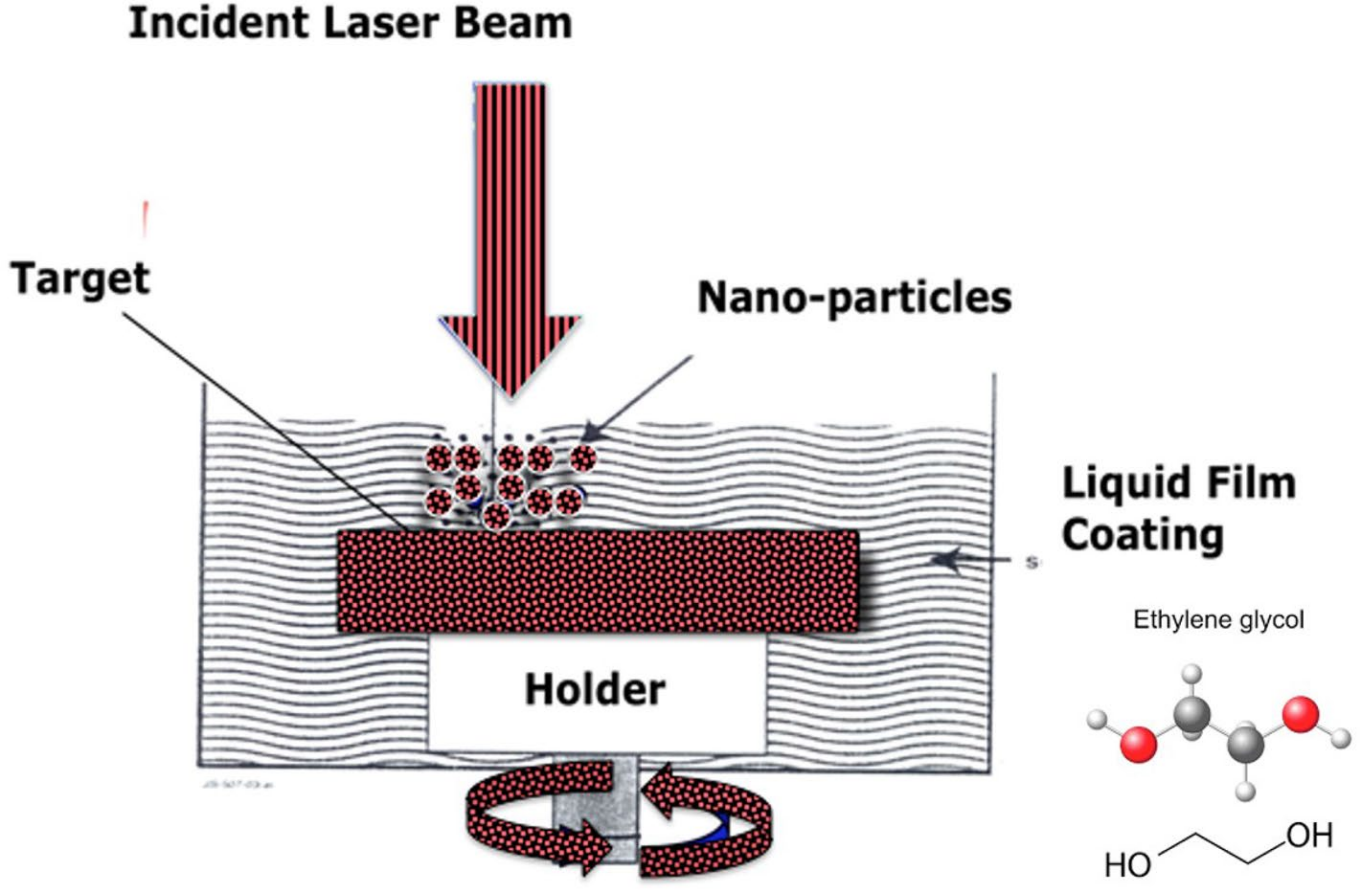
Simplified configuration of the laser liquid solid interaction set-up. The normal incidence laser beam is focused on the metallic target coated with Ethylene Glycol.

As mentioned above, the synthesis of the AgNPs/GNs/EG nanofluid was performed in 2 steps utilizing the so-called Laser Liquid Solid interaction (LLSI) also known as pulsed laser ablation in liquids (PLAL). Indeed, in parallel with the standard well-established vacuum pulsed laser deposition (PLD) technology dedicated to the synthesis of nano-structured and nanophased materials in the form of thin film coatings, LLSI/PLAL is a hybrid chemical–physical laser based approach, promoted for the synthesis of metallic and oxide nanoparticles in a form of colloidal nanosuspensions^38,39,40^. This nanoparticles’ synthesis technique is based on laser—solid interaction with the target immersed in a liquid or covered by a protective liquid layer (Fig. 3). The advantage of this approach is due to the combination of two major effects namely, the standard ablation process due to the laser-matter interaction and the acoustic process caused by explosion of the native gas bubbles during the local overheating at the target-liquid interface (in this case, EG vapor, T^V^_EG_1 ~ 97.3 °C). As shown in Fig. 3, the principle of the LLSI/PLAL technique used in the current study consisted of a short interaction (nanosecond regime) between a pulsed focused laser beam impinging onto graphite and Ag targets during the 1st and 2nd phases, respectively, both immersed in ethylene glycol while all enclosed in a stainless-steel container. Each of the immersed rotating targets (~ 120 rpm) is irradiated with pulses from the incident Nd-YAG laser beam and hence clusters of the ablated materials are generated. During the early stages of ablation, nanoparticles form as a result of clustering during self-assembly and subsequently dispersed within the ethylene glycol fluid which also acts as an effective surfactant. Yet, EG is not a polar molecule because it is symmetrical, it contains internal dipoles, yet its net dipole moment is zero. Its internal dipoles oppose each other. Ethylene glycol contains both polar and nonpolar parts. Hence, it could be expected that a chemical interaction of freshly ablated GNs flakes with host fluid molecules EG (C_2_H_6_O_2_) could take place.

**Figure 4:**
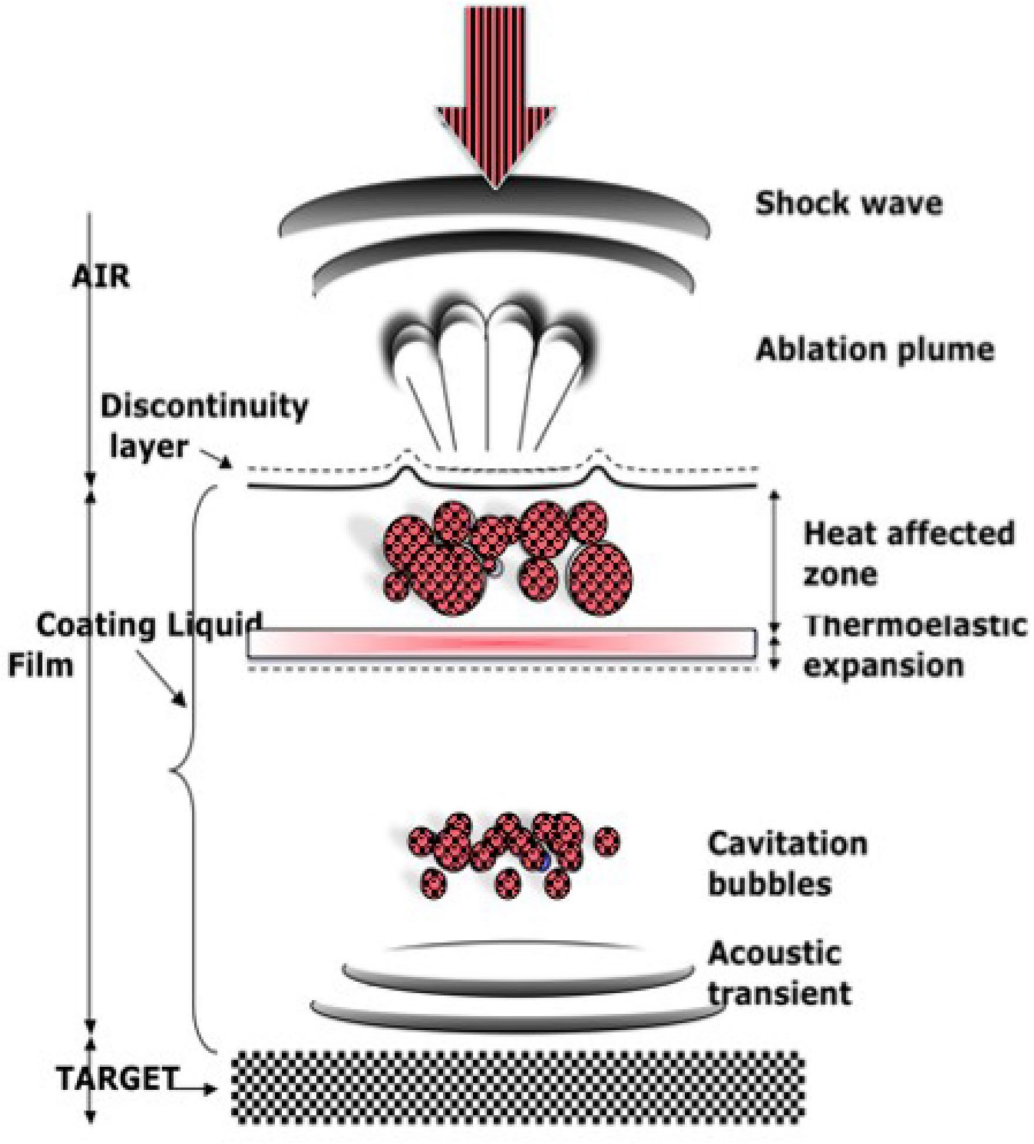
Possible mechanisms causing the ablation in laser liquid solid interaction. Cavitation and implosion of the formed vapor bubbles.

The real mechanisms of ablation are not discussed in detail as these, major physical phases are well established. As depicted in Fig. 4, the irradiation of a target through a liquid layer can drive several interrelated phenomena, namely, acoustic transient, followed by a thermoelastic expansion, which induces a bubbles’ cavitation phenomenon. The energy absorption at the target-liquid interface increases the local ethylene glycol temperature and the subsequent thermal expansion of the heated volume produces thermoelastic-stress waves^38,39,40^. As a result, a vapor phase in the form of bubbles is formed. As in the case of H_2_O, acoustic excitations are then induced by the dynamics of the vapor plume as well as by the locally overheated metallic target^38,39,40^. The effect of the two acoustic excitations provokes the implosion of gas bubbles inducing ablation at the surface of the melted metallic target at the “focus point of the incident laser beam” in the case of Ag target and causing a removal of graphene sheets in the case of graphite target. As the ablated particles are in the nano-size range, this suggests that the bubbles are succumb to a positive external pressure before the implosion. Excluding the laser beam parameters, one should expect that the size of the synthesized nanoparticles by this laser liquid interaction, would depend on the bubbles nucleation rate and their implosion cycles^38,39,40^. Taking into account the different mechanisms mentioned above, one is led to conclude that LLSI/PLAL is likely to be governed by a photo-acoustic energy transfer phenomenon. The corresponding maximum energy fluence of the Nd-YAG was limited to ~ 85 mJ/pulse. The ablation time varied from 5 to 30 min in order to control the volume concentration of the nanoparticles within the ethylene glycol based nanofluid. The temperature of the EG coating fluid layer/ target interface is certainly above the ethylene glycol boiling point of 197.3 °C (470.4 K). The estimated induced temperature at the point where the laser pulse impinges on the target submerged in the EG is estimated to be around T_int_ = (hc/k_B_λ) ~ 14.2 × 10^4^ K, significantly above the melting (1,236 K: Ag) and vaporization (2,223 K: Ag, 3,773; Graphite) temperatures of the Ag and graphite targets. Accordingly, and in view of the above considerations, one could expect that while the Ag NPs follow the standard ablation mechanisms to form silver particle clusters as a result of agglomeration, the graphene sheets would, likely, form via acoustic effects mechanisms. More precisely, single or multiple sheets of graphene should be pulled out of the graphite target.

From experimental point of view, graphite and silver targets (both 99.9% purity as well as the ethylene glycol (EG anhydrous 99.8%) used in this study were of chemical grade supplied by Sigma-Aldrich. In phase One, a graphite target with (< Ø >  ~ 10 mm in diameter and 3 mm thick was placed at the bottom of the rotating cylindrical container filled with 20 mL of EG. The spectra physics GCR 4–10 Nd:YAG laser operating at the fundamental wavelength (λ ~ 1.064 nm), pulse duration of 12 ns, repetition rate of 10 Hz and an output energy of ~ 85 mJ/pulse was focused through a convex lens with a focal length of f ~ 300 mm to produce graphene nanosheets nanofluid in EG with the processing time of 5–30 min. In phase two, the graphite target was replaced by the silver target(< Ø >  ~ 5 mm diameter and 1 mm thick). The ablation time was fixed at 20 min. After ablating Ag, the Ag target was removed and the AgNPs/GNs/EG nanofluid was collected for further analysis. For comparison purposes and as reference sample, pure graphene-EG and Ag-EG nanofluids were prepared in similar conditions. Hence pure Ag, pure graphene, and Ag-graphene nanofluids will be referenced as AgNPs-EG, GNs-EG, and Ag-GNs-EG, respectively.

### Materials and nanofluid characterization

The crystalline structure of AgNPs/GNs/EG nanofluid coated on a glass substrate was investigated by X-ray diffraction (XRD) using a SmartLab (Rigaku) diffractometer with a CuKα_1_ radiation (λ = 1.5406 Å) with a scanning angular step of 0.2° s^−1^ over angular range 2θ of 20°–90°. The morphology of AgNPs/GNs/EG nanofluid was studied by using a Leo-Stereo high-resolution scanning electron microscope (HRSEM). Identification and characterization of the functional groups on the nanoparticles of the nanofluid was carried out using a Perkin Elmer ATR-FTIR spectrometer within the spectral range of 500–4,000 cm^−1^. The Raman studies were carried out using a WITEC Alpha 300 Confocal Raman system operating with the second harmonic of a Nd:YAG laser (λ_Exc_ = 532 nm) as the excitation source. The size and morphology of the nanoparticles in suspension within the nanofluid were determined using a JEOL JEM 2010F microscope. The thermal conductivity of the fabricated AgNPS/GNs/EG nanofluid was investigated by a simplified transient hot-wire technique.

### Morphological investigations

**Figure 5:**
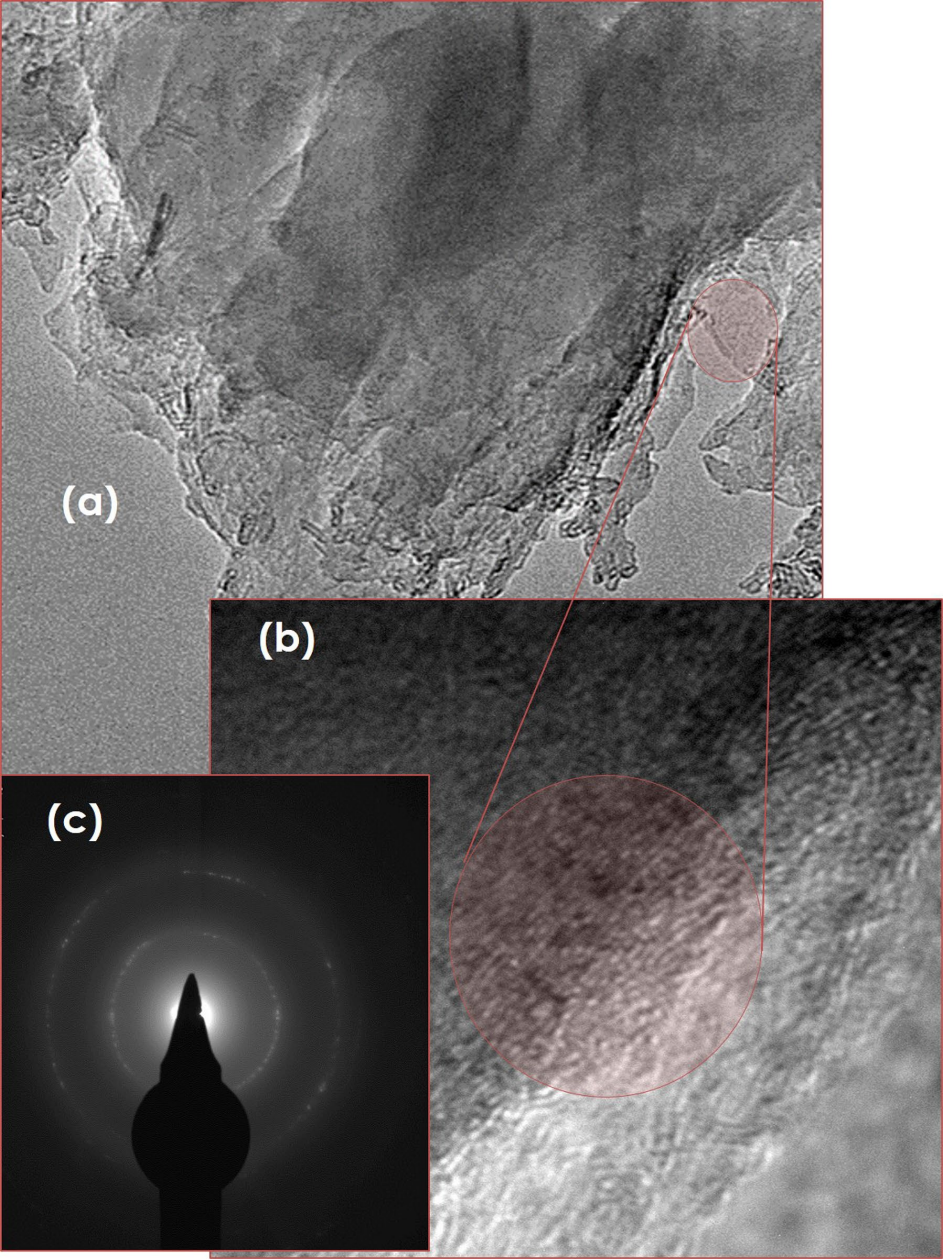
(**a**, **b**) High Resolution Transmission Electron Microscopy Images of the Graphene sheets and (**c**) its corresponding Selected Area Electron Diffraction of the GNs-EG samples.

**Figure 6:**
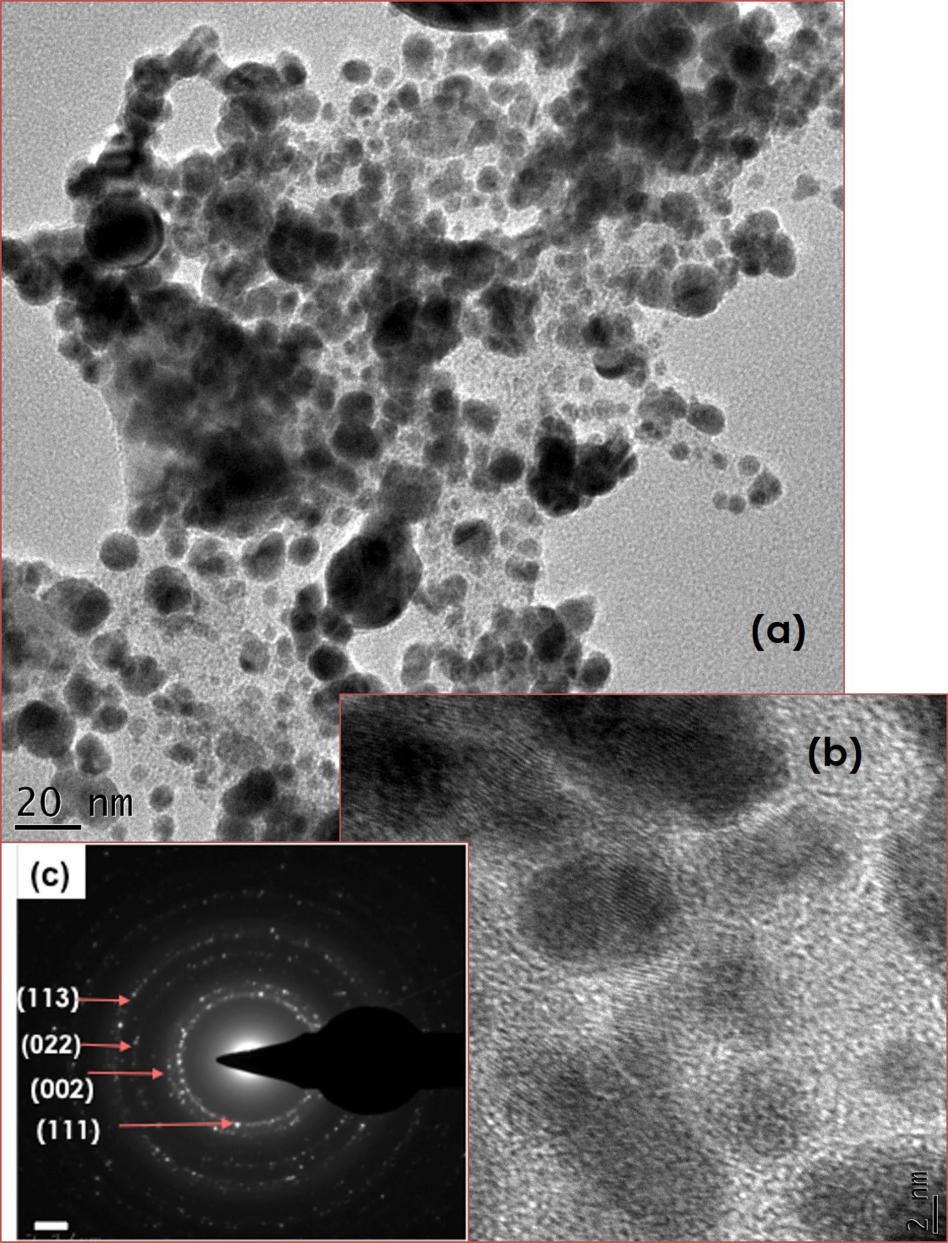
(**a**, **b**) High Resolution Transmission Electron Microscopy Images of the Silver Nanoparticles and (**c**) their corresponding Selected Area Electron Diffraction of the AgNPs-EG samples.

In Fig. 5a, b show the low and high magnification micrographs of pure graphene suspensions (GNs-EG) indicating that, generally, the graphene consists of several sheets with mainly (1–210) and (1–110) crystallographic orientations as shown in the selected area electron diffraction pattern of Fig. 5c. Figure 6 shows similar observations carried out on pure Ag nanosuspensions (AgNPs-EG sample). The Ag nanoparticles are quasi-spherical in shape in general with a likely bimodal size distribution. Such a bimodal distribution is centered at 〈Ø〉≈16.2 nm and 47.6 nm, respectively Fig. 6a. They are non-agglomerated with border to border average distance of about 〈ξ〉 ≈ 3 nm and 〈ξ〉 ≈ 12 nm between neighbouring small and neighbouring large Ag nanoparticles, respectively. Such relatively small distances between the Ag nanoparticles (considering the Stokes Einstein approximation), the average diffusion coefficient of the Ag nanoparticles within the ethylene glycol D_Ag_ = k_B_T /6πη〈Ø/2〉 at room temperature is about 16.710^–16^ m/s^2^ and 3.2310^–16^ m/s^2^, respectively, (η_EG_ = 14 (mPa.s)). Such low diffusion coefficients, relative to the above estimated border to border separation distances of 〈ξ〉 ≈ 3 nm and 〈ξ〉 ≈ 12 nm seem to indicate that the AgNPs are unlikely to agglomerate at room temperature. From crystallographic point of view, the selected area electron diffraction (SAED pattern) of Fig. 6c indicates that the Ag nanoparticles are polycrystalline in nature with no privileged texture.

**Figure 7:**
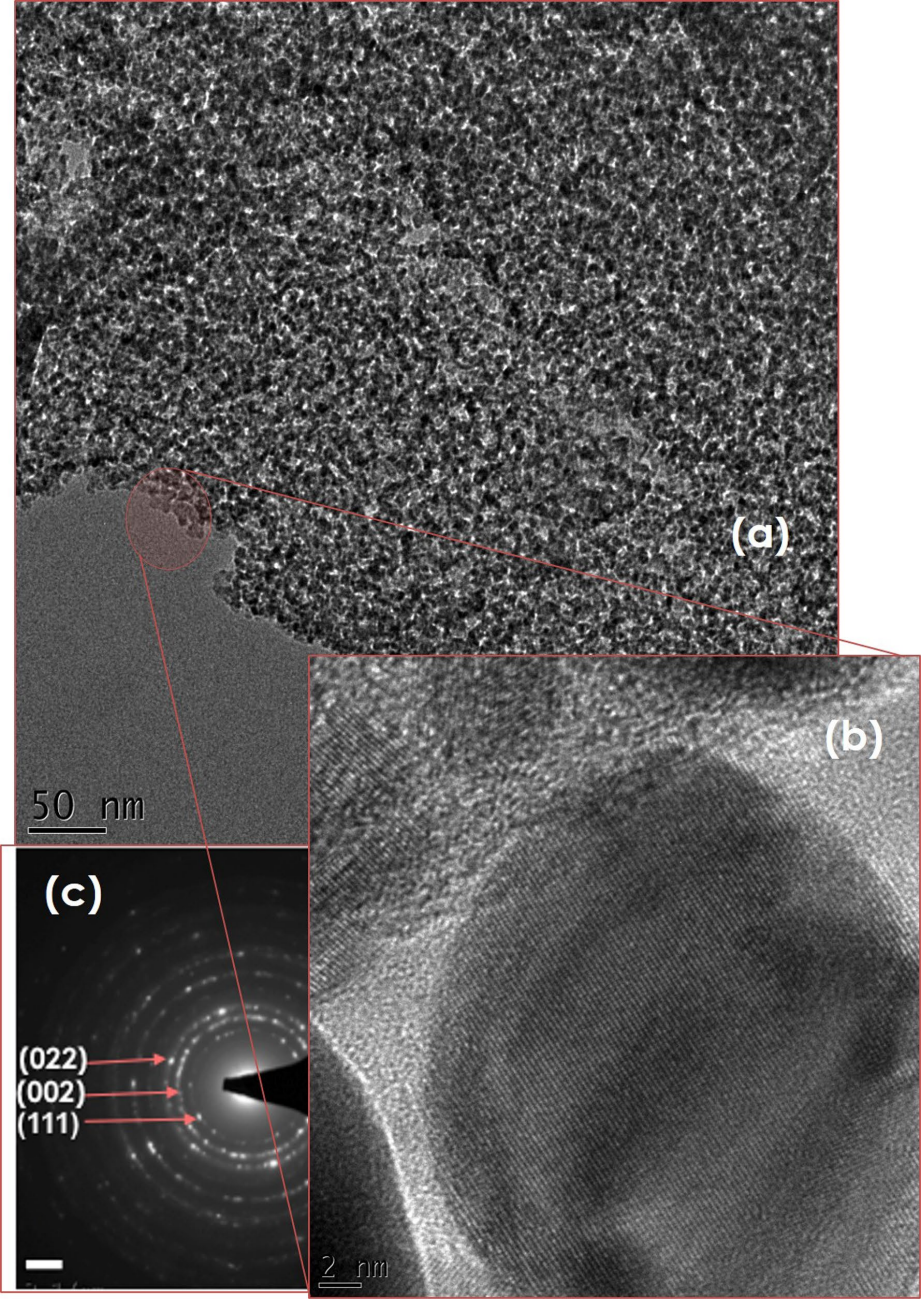
(**a**, **b**) High Resolution Transmission Electron Microscopy Images of the Silver Nanoparticles densely decorating Graphene sheets and (**c**) their corresponding Selected Area Electron Diffraction in the AgNPs-GNs-EG samples.

Figure 7a, b shows the low and high magnification of the transmission electron microscopy of the AgNPs-GNs-EG sample. As one can notice, the 2-D GNs are densely decorated with Ag NPs. Such a densification of (Ag NPs) could be due to their polarity and hence facilitating their anchorage onto the surface of the 2D GNs carpets. Similar phenomenon was observed and reported by Wook Kang et al*.*^41^, Vi and Lue et al*.*^42^, as well as Zhang et al*.*^43^. It is observed that such an anchoring process of the Ag NPs onto the GNs carpets is stable with time, confirming the homogeneous dispersion and the stability with time of such an anchoring. We also investigated the nature of such Ag NPs liaison onto GNs in order to understand its physical/chemical nature. The high-resolution transmission electron microscopy observations Fig. 7b indicate that the Ag NPs are crystalline with different crystallographic orientations and textures as evidenced by the selected area electron diffraction of Fig. 7c.

### Elemental analysis

**Figure 8:**
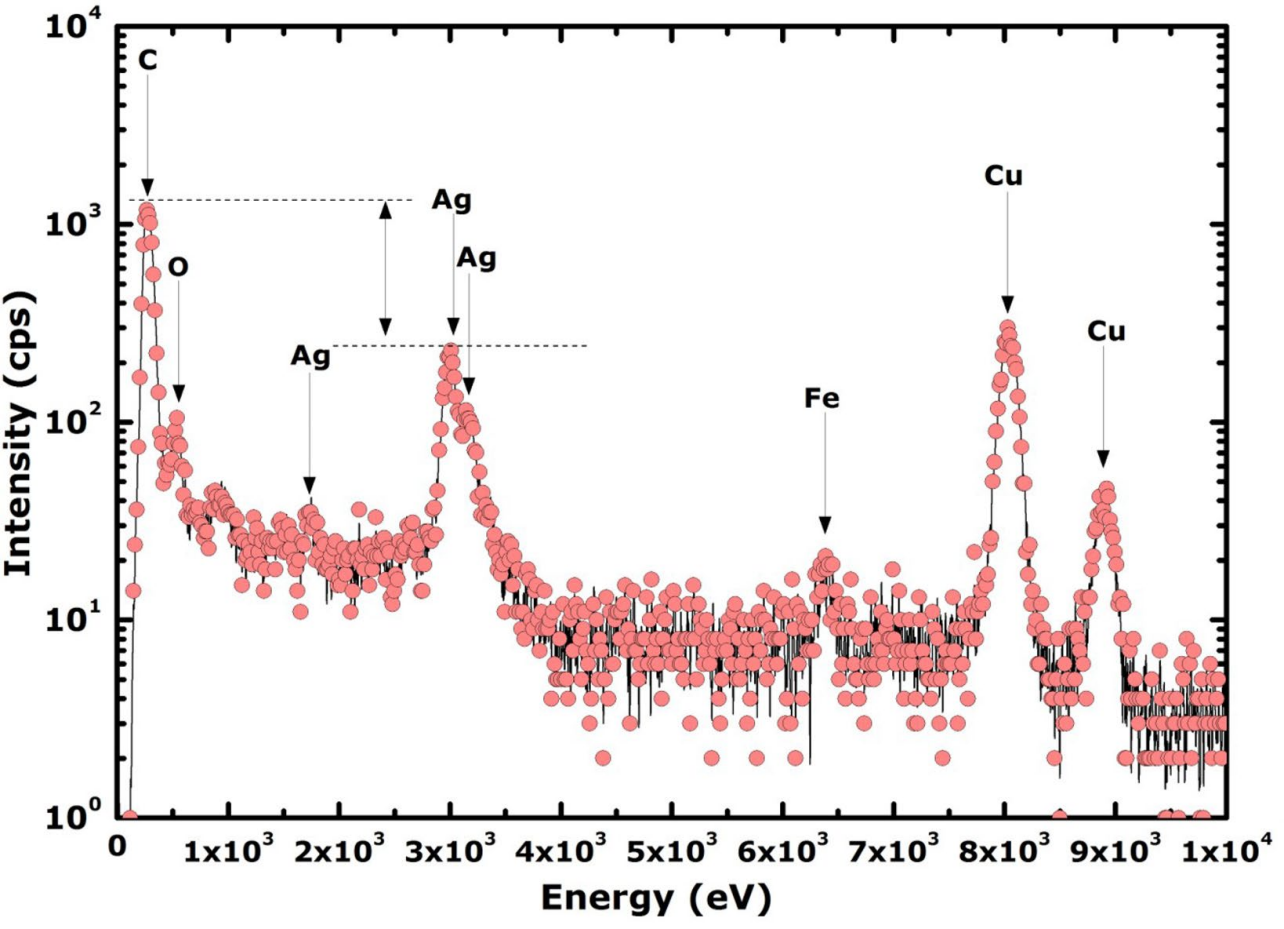
Typical Elemental Energy Dispersive X-rays Spectroscopy spectrum of AgNPs-GNs-EG samples.

Figure 8 shows a typical energy dispersive spectroscopy (EDS) elemental spectrum of the AgNPs-GNs-EG nanofluid. Several channel peaks of Ag were observed. The Cu peaks at higher channels originate from the Cu grid used for the HRSEM observations. Yet, at the background level, the Fe peak located at vicinity of ~ 6.5 keV channel is likely to be an impurity from either the graphite or Ag used targets (N3 chemical purity). The C intense peak at the lowest channel is attributed to the graphene sheets as free ‘C’ coating and free Cu grid are used for these observations. Likewise, it is noteworthy to point out that the oxygen peak at lower channel ~ 0.5 keV is likely to be attributed to ‘O’ originating from the host fluid EG. More precisely, this oxygen is probably bound on the graphene sheets’ surface explaining the substantial stability of the 2-D graphene carpets within the host EG fluid.

### Vibrational investigations

**Figure 9:**
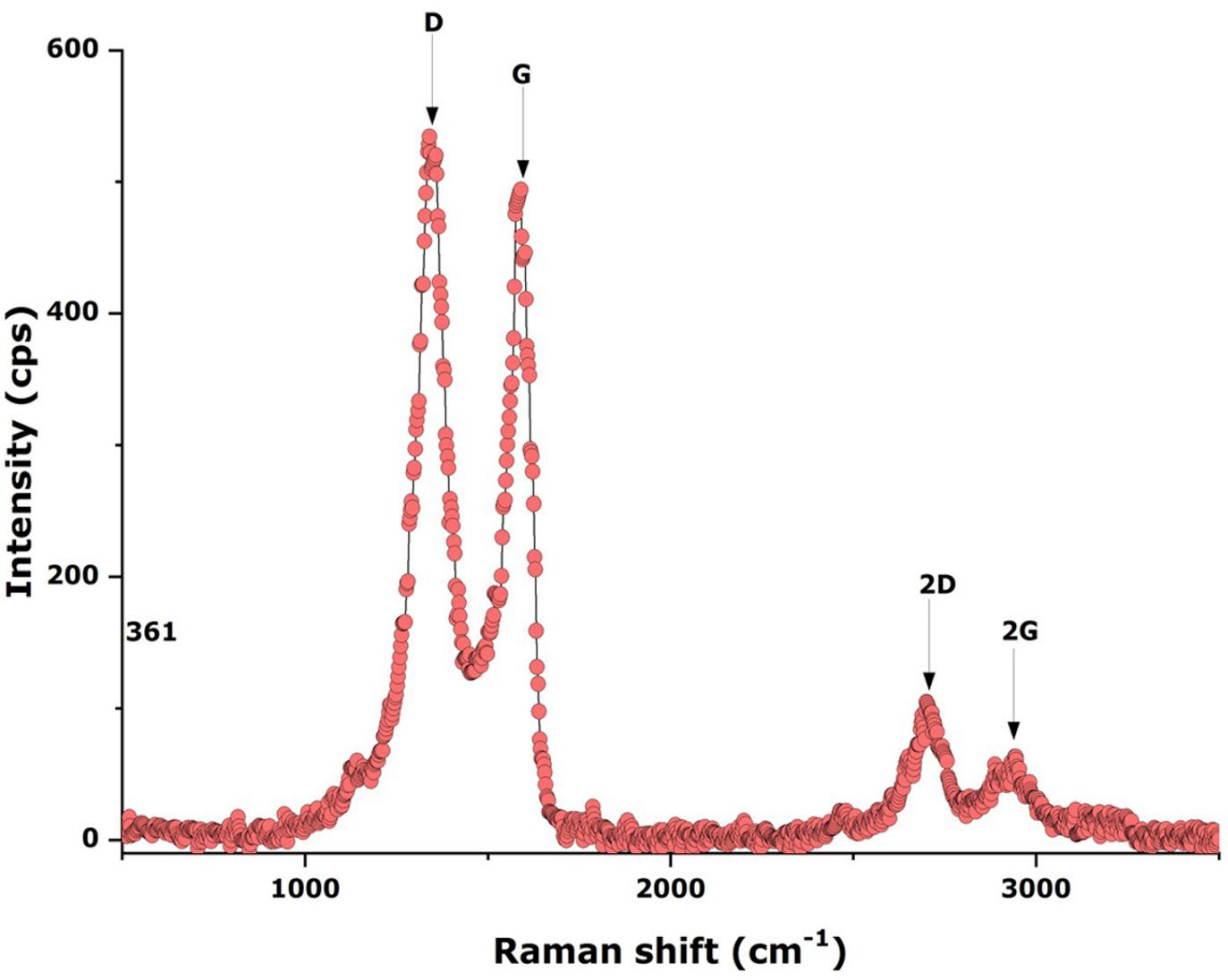
Typical room temperature Raman spectrum of AgNPs-GNs-EG samples.

Figure 9 shows the room temperature Raman spectrum of the AgNPs-GNs-EG within the spectral range of 500–3,500 cm^−1^. One can distinguish clearly the D (1,343 cm^−1^) and G (1,580 cm^−1^) bands as well as the 2D and the 2G bands representative of a few layered graphene. As established, the D peak originates from TO phonon mode near K points in the Brillouin zone. This peak is only activated by structural defects as well as second order Raman scattering process through the intervalley double resonance and demonstrates the crystallinity of the sample^44^ in congruence the previous observations. The Raman spectra also show the first order Raman peak located at 1,580 cm^−1^ denoted by G band originating from the doubly degenerate zone center phonon E_2g_ mode which corresponds to the C–C stretching modes^43^. The peak appearing at 2,680 cm^−1^ is the second order Raman peak, denoted by 2D and overtones D mode^44^. The intensity of the D peak (1,343 cm^−1^) intensity is higher than that of the G peak with an intensity ratio (I_D_/I_G_) of about ~ 1% pointing to a high crystallinity within the graphene nanosheet^45^.

### Crystallographical analysis

**Figure 10:**
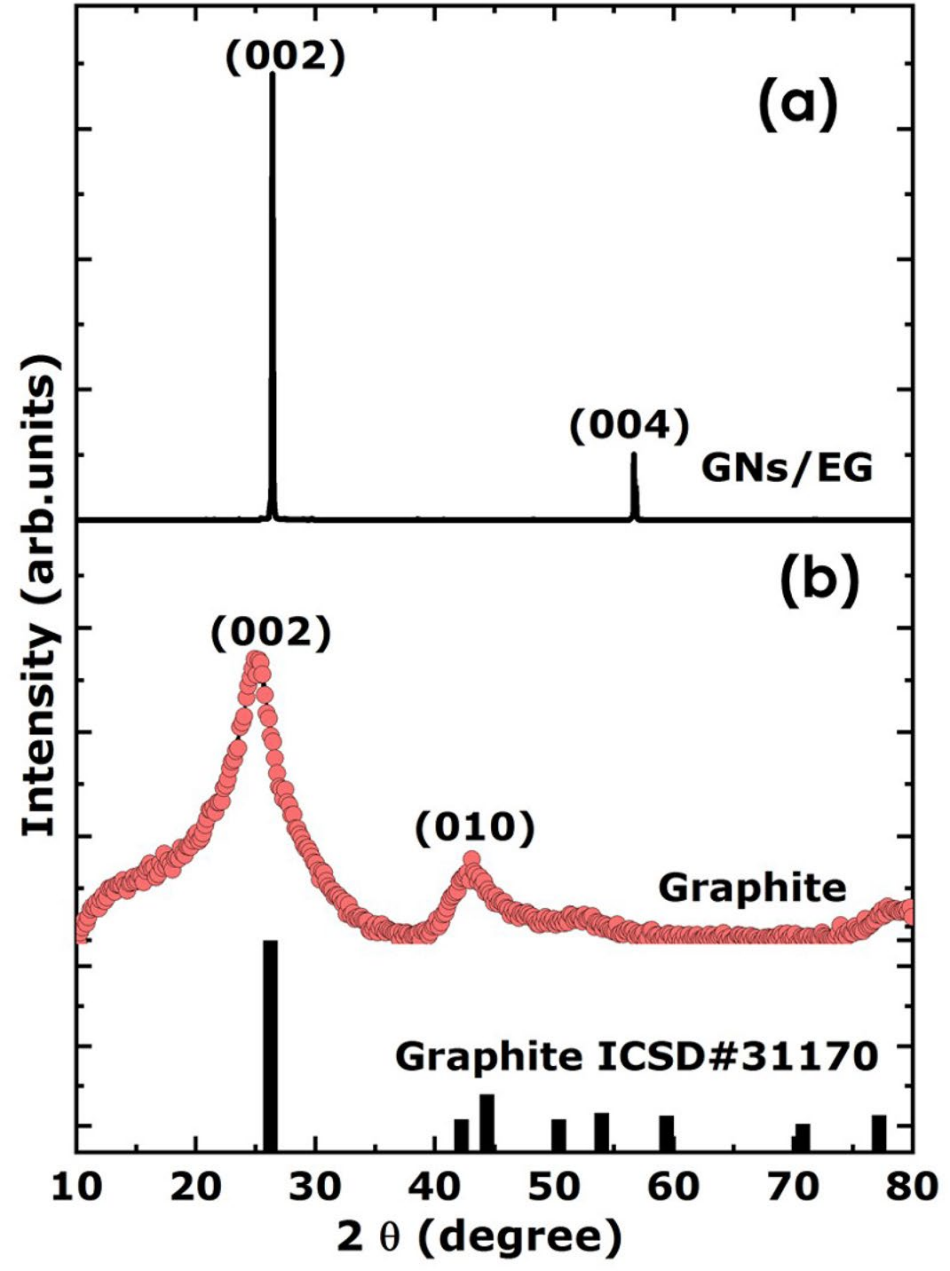
(**a**) Typical room temperature X-rays diffraction spectrum of GNs-EG samples (**b**) and that of the pure graphite target.

Figure 10a reports the room temperature X-rays diffraction (XRD) spectrum of the GNs-EG; and that of the corresponding graphite target as shown in Fig. 10b. While the GNs-EG sample exhibits mainly four intensity weak broad band Bragg peaks corresponding to the (002), (010), (004) and (110), the graphite target displays two sharp intense Bragg peaks; (002) and its corresponding second order (004). Equally, it is important to mention the absence of any signature of free graphene oxide suggesting, a priori, no chemical reaction between the graphene formed nanosheets and the surrounding fluid C_2_H_6_O_2_ if any at least at the 10% limit of detection of XRD technique limitation. This does not provide evidence of a potential physisorption or chemisorption at the surface of the 2-D Graphene sheets of Oxygen from the surrounding host fluid C_2_H_6_O_2_ molecules as corroborated by the previous EDS observations.

### Optical spectroscopy investigations

**Figure 11:**
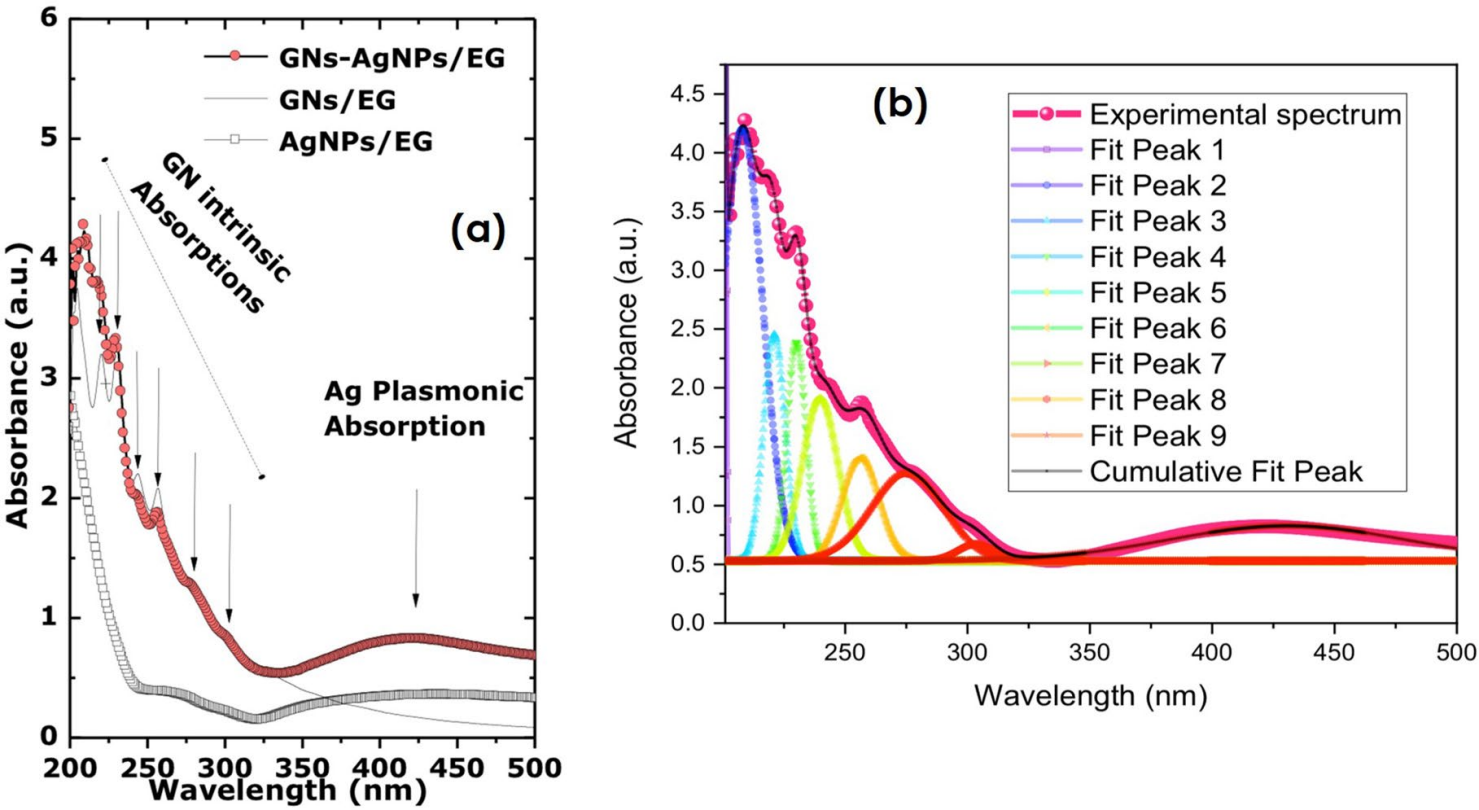
(**a**) Typical Optical Absorbance of AgNPs-GNs-EG samples compared to that of GNs-EG and AgNPs-EG profiles; (**b**) Cumulative fit peak of the various π−π*, n–p* transitions, Graphene, Graphene oxide absorbance peaks and the Ag plasmonic absorbance.

Figure 11a reports the room temperature optical absorbance of the various nanofluids (AgNPs-EG, GNs-EG and AgNPs-GNs-EG) in the spectral range of 200–500 nm. If one focuses only on the AgNPs-GNs-EG spectrum, one can distinguish the specific plasmon peak of the Ag NPs centered at about 430 nm^35^. This wide plasmon peak implies the non-agglomeration of the Ag nanoparticles as well as their small average size. Based on *Mie Scattering Theory (MST)*, where the width at half maximum of the plasmon peak is related to the average size via the relation: $\left\langle {\Delta \omega_{{{1}/{2}}} } \right\rangle$〈Δω_1/2_〉 = γ_0_ + Av_F_/〈Ø〉 (equivalent to: $\left\langle {\Delta \lambda_{{{1}/{2}}} } \right\rangle$ = λ^2^/hc) (γ_0_ + Av_F_/〈Ø〉)), where 〈Ø〉 is the size of nanoparticle, A = 3/4 for silver, γ_0_ is the velocity of bulk scattering (510^12^ deg s^−1^), v_F_ is the Fermi velocity (for silver v_F_ = 1.3910^6^ deg m/s), the corresponding average size is about 〈Ø〉 ≈ 21.7 nm in the size range of those observed in the previous HRTEM observations. In the spectral range of 200–300 nm, there is a strong absorbance peak centered at 230 nm with a broad shoulder in both GNs-EG and AgNPs-GNs-EG samples as shown in the Fig. 11b. These are attributed to the π−π* and to the n-p* transitions, respectively^35,46^. In addition to the wide plasmon peak of the AgNPs, and the proper GNs absorbances, there is an additional set of quasi sharp absorbances in the AgNP-GNs-EG sample. These various substructure absorption peaks within the UV spectral range of 200—350 nm, are simulated and are summarized in Table 1. The strong absorption peak centered at 230 nm present in both AgNPs-GNs-EG and GNs-EG absorbances and not in the AgNPs ‘s profile is attributed to the π → π* transitions of aromatic C=C bonds while the shoulder within 250–350 nm is attributed to the n → π*^47^. Likewise, both AgNPs-GNs-EG and GNs-EG absorbances exhibit a band at 261 nm which is attributed to pure graphene, while the 273 nm absorbance indicates the restoration of extensive conjugated sp^2^ carbon network^48,49,50,51^. The peaks at 221 nm and 430 nm are attributed to graphene oxide as reported previously^52^. This graphene oxide seems to indicate a significant chemical binding between the graphene sheets and the host fluid molecules of C_2_H_6_O_2_ as stipulated in the previous EDS observations.

**Table 1:** Major characteristics of the various observed peaks within the absorbance spectrum of the AgNPs-GNs-EG nanofluid.

	Peak-1	Peak-2	Peak-3	Peak-4	Peak-5	Peak-6	Peak-7	Peak-8	Peak-9 (Ag-plasmon)
λ (nm)	201.50	208.06	221.08	230.12	239.77	256.40	275.51	303.56	430.70
Δλ/(nm)	0.53	16.27	8.78	7.73	14.3	15.03	29.37	14.80	96.04

### Thermal conductivity studies

**Figure 12:**
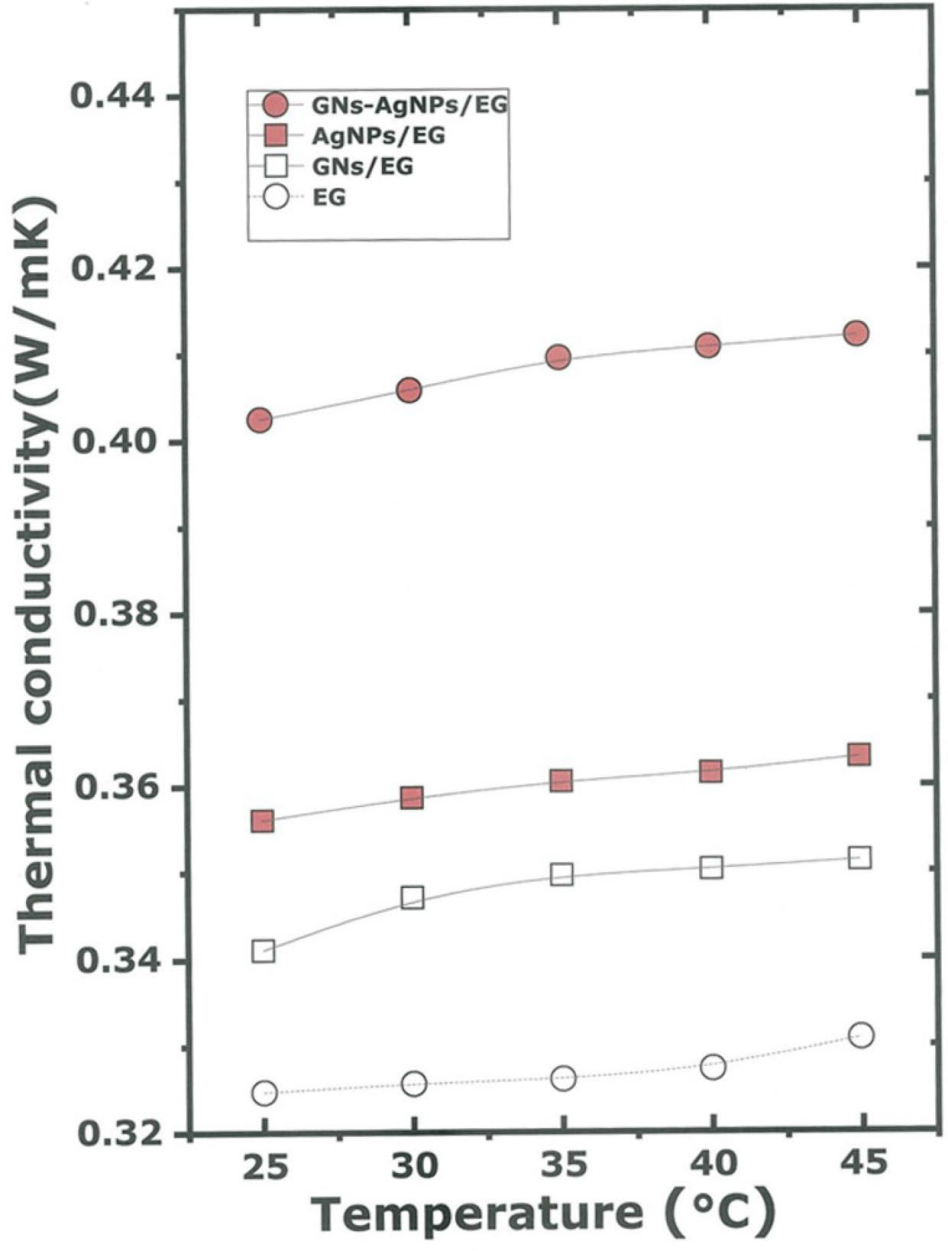
Thermal conductivity of the various nanofluids compared to the pure EG in the temperature range of 25–45 °C.

Figure 12 shows a plot of the thermal conductivity versus temperature in the range 25–45 °C for the materials under test. At 25 °C, the thermal conductivity measured by the standard Pt wire methodology is about 0.325, 0.340, 0.355 and 0.410 W/mK for pure EG, AgNPs-EG, GNs-EG and AgNPs-GNs-EG respectively. The thermal conductivity measurement for AgNPs-GNs-EG nanofluid was found to be 32.3% higher than that for pure EG. The thermal conductivity was found to be temperature independent for EG, AgNPs-EG, GNs-EG nanofluids in the temperature range under test^56,57^. However, for AgNPs-GNs-EG nanofluid there was a slight increase with temperature with a slope of about 7.0 × 10^−4^ W/mT^2^. At 45 °C, the thermal conductivity reaches 0.414 W/mK. When compared with the results, reported in literature on dispersed graphene in ethylene glycol summarized as shown in Table 2, and on the thermal conductivity enhancement of EG based nanofluids^47,51,52,54,55^ the current enhancement value of 32.3% seems to be the highest so far.

**Table 2:** Reported thermal conductivity enhancement of graphene—EG based nanofluids.

References	Host fluid	Configuration	Temperature range (°C)	Thermal conductivity enhancement (%)
Baby and Ramaprabhu^55^	EG	Exfoliated graphene	25–50	4–7%
Baby and Ramaprabhu^52^	EG	Hydrogen exfoliated graphene	25–50	1–7.5%
Lee and Rhee^46^	EG	Graphene nanoplatelets	10–90	Up to 32%
Shende and Sundara^53^	EG	Nitrogen doped graphene-MNT	25–50	Up to 15.1%
Wang^55^		Exfoliated graphene	25–65	15.5–18.6%
Mbambo et al. (present work)	EG vapor—T^V^_EG_1 ~ 97.3 °C	Ag—decorated graphene nanocomposites	25–45	~ 32.3%

## Conclusions

In this contribution, we caused a multicomponent nanofluid based on silver NPs densely decorated with 2D graphene sheets was dispersed homogeneously with ethylene glycol as host base fluid sample. Such a stable multicomponent nanofluid was synthesized by laser liquid solid interaction in a two steps approach. Such a technology approach allows a significant anchorage of the AgNPs onto the graphene 2D sheets. The thermal conductivity of such nanofluids was measured to be 0.414 W/mK corresponding to a remarkable enhancement of the order of 32.3% compared with ethyl glycol. This is the highest reported value for a graphene based nanofluid. Further studies are required in order to understand the reason for the high anchoring of the Ag NPs onto the GNs sheets as well as the possibility of engineering Ag nanorods decorated GNs-EG based nanofluids.
